# Supplementary material for: Pyruvate Dehydrogenase Kinase 1 inhibition mediated oxidative phosphorylation enhancement in cartilage promotes osteoarthritis progression
Source: BMC Musculoskelet Disord. 2023 Jul 20;24:597. doi: 10.1186/s12891-023-06585-6 (PMC10357736; doi:10.1186/s12891-023-06585-6)
Supplement: Supplementary file 1 — Additional file 1. [file 12891_2023_6585_MOESM1_ESM.zip › BP.Gsea.1653623667859/GOBP_ACTIN_MYOSIN_FILAMENT_SLIDING.html]

Details for gene set GOBP\_ACTIN\_MYOSIN\_FILAMENT\_SLIDING[GSEA]

|  || Dataset | 55457\_collapsed\_to\_symbols.cls.cls #OA\_versus\_normal.cls.cls #OA\_versus\_normal\_repos |
| Phenotype | cls.cls#OA\_versus\_normal\_repos |
| Upregulated in class | OA |
| GeneSet | GOBP\_ACTIN\_MYOSIN\_FILAMENT\_SLIDING |
| Enrichment Score (ES) | 0.509424 |
| Normalized Enrichment Score (NES) | 1.5176207 |
| Nominal p-value | 0.04988662 |
| FDR q-value | 0.3502147 |
| FWER p-Value | 1.0 |
Table: GSEA Results Summary

  

Fig 1: Enrichment plot: GOBP\_ACTIN\_MYOSIN\_FILAMENT\_SLIDING      
 Profile of the Running ES Score & Positions of GeneSet Members on the Rank Ordered List

  

| SYMBOL | TITLE | RANK IN GENE LIST | RANK METRIC SCORE | RUNNING ES | CORE ENRICHMENT || 1 | MYL6B | myosin light chain 6B [Source:HGNC Symbol;Acc:HGNC:29823] | 815 | 0.565 | 0.0862 | Yes |
| 2 | ACTC1 | actin alpha cardiac muscle 1 [Source:HGNC Symbol;Acc:HGNC:143] | 2331 | 0.323 | 0.0540 | Yes |
| 3 | TNNC1 | "troponin C1, slow skeletal and cardiac type [Source:HGNC Symbol;Acc:HGNC:11943]" | 2396 | 0.315 | 0.1324 | Yes |
| 4 | MYH2 | myosin heavy chain 2 [Source:HGNC Symbol;Acc:HGNC:7572] | 2459 | 0.307 | 0.2088 | Yes |
| 5 | MYL1 | myosin light chain 1 [Source:HGNC Symbol;Acc:HGNC:7582] | 2491 | 0.303 | 0.2867 | Yes |
| 6 | MYH6 | myosin heavy chain 6 [Source:HGNC Symbol;Acc:HGNC:7576] | 2555 | 0.297 | 0.3603 | Yes |
| 7 | MYLPF | "myosin light chain, phosphorylatable, fast skeletal muscle [Source:HGNC Symbol;Acc:HGNC:29824]" | 2816 | 0.268 | 0.4111 | Yes |
| 8 | MYH8 | myosin heavy chain 8 [Source:HGNC Symbol;Acc:HGNC:7578] | 3119 | 0.238 | 0.4506 | Yes |
| 9 | MYH7 | myosin heavy chain 7 [Source:HGNC Symbol;Acc:HGNC:7577] | 3161 | 0.234 | 0.5094 | Yes |
| 10 | MYBPC3 | myosin binding protein C3 [Source:HGNC Symbol;Acc:HGNC:7551] | 3926 | 0.162 | 0.4930 | No |
| 11 | TPM1 | tropomyosin 1 [Source:HGNC Symbol;Acc:HGNC:12010] | 4220 | 0.135 | 0.5061 | No |
| 12 | MYH4 | myosin heavy chain 4 [Source:HGNC Symbol;Acc:HGNC:7574] | 4702 | 0.096 | 0.4941 | No |
| 13 | TNNT2 | "troponin T2, cardiac type [Source:HGNC Symbol;Acc:HGNC:11949]" | 7441 | -0.114 | 0.3118 | No |
| 14 | MYH3 | myosin heavy chain 3 [Source:HGNC Symbol;Acc:HGNC:7573] | 8258 | -0.176 | 0.2949 | No |
| 15 | MYL6 | myosin light chain 6 [Source:HGNC Symbol;Acc:HGNC:7587] | 9188 | -0.247 | 0.2881 | No |
Table: GSEA details [plain text format]

  

Fig 2: GOBP\_ACTIN\_MYOSIN\_FILAMENT\_SLIDING      
 Blue-Pink O' Gram in the Space of the Analyzed GeneSet

  

Fig 3: GOBP\_ACTIN\_MYOSIN\_FILAMENT\_SLIDING: Random ES distribution      
 Gene set null distribution of ES for **GOBP\_ACTIN\_MYOSIN\_FILAMENT\_SLIDING**
